# Supplementary material for: Faecalibacterium duncaniae Mitigates Intestinal Barrier Damage in Mice Induced by High-Altitude Exposure by Increasing Levels of 2-Ketoglutaric Acid
Source: Nutrients. 2025 Apr 19;17(8):1380. doi: 10.3390/nu17081380 (PMC12030221; doi:10.3390/nu17081380)
Supplement: Supplementary file 1 [file nutrients-17-01380-s001.zip › nutrients-3559680-supplementary.pdf]

Table S1. Primers used in this study.

Targets	Primer	Sequence (5′–3′)	Reference
Faecalibacterium	F	AGATGGCCTCGCGTCCGA	(1)
	R	CCGAAGACCTTCTTCCTCC	
Nfkbia	F	GGCAATCATCCACGAA	
	R	CCAAGGCAGATAAGAACTA	
Fos	F	AGGCAGAACCCTTTGA	
	R	GGTGACCACGGGAGTA	
GAPDH(4)	F	ATCAACGACCCCTTCATTGACC	(2)
	R	CCAGTAGACTCCACGACATACTCAGC	

References

(1) A. Cardoneanu, S. Cozma, C. Rezus, F. Petrariu, A. M. Burlui and E. Rezus, Characteristics of the intestinal microbiome in ankylosing spondylitis, Exp Ther Med, 2021, 22, 676.

(2) H. G. Kim, P. F. Hillman, Y. J. Lee, H. E. Jeon, B. K. Lim and S. J. Nam, Caboxamycin Inhibits Heart Inflammation in a Coxsackievirus B3-Induced Myocarditis Mouse Model. Viruses, 2024, 16, 677.

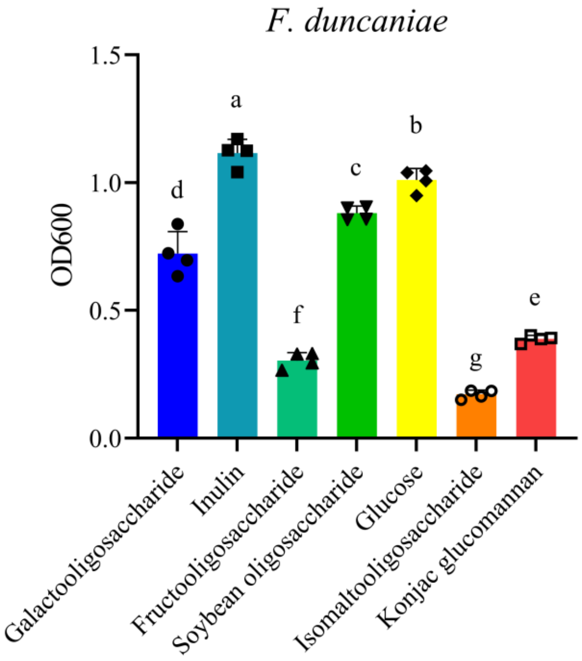

Figure S1. The effects of six prebiotics on the growth of *F. duncaniae* were analyzed using one-way ANOVA. Different letters indicate significant differences. Data are presented as means ± SD (n = 4).
